# Complement deposition at the neuromuscular junction in seronegative myasthenia gravis

**DOI:** 10.1007/s00401-020-02147-5

**Published:** 2020-03-10

**Authors:** Sarah Hoffmann, Lutz Harms, Markus Schuelke, Jens-Carsten Rückert, Hans-Hilmar Goebel, Werner Stenzel, Andreas Meisel

**Affiliations:** 1 Department of Neurology and NeuroCure Clinical Research Center, Berlin Institute of Health (BIH), Charité—Universitätsmedizin, Freie Universität Berlin, Humboldt-Universität Zu Berlin, Berlin, Germany; 2 Department of Neuropediatrics and NeuroCure Clinical Research Center, Berlin Institute of Health (BIH), Charité—Universitätsmedizin, Freie Universität Berlin, Humboldt-Universität Zu Berlin, Charitéplatz 1, 10117 Berlin, Germany; 3 Department of Thoracic Surgery, Berlin Institute of Health (BIH), Charité—Universitätsmedizin, Freie Universität Berlin, Humboldt-Universität Zu Berlin, Berlin, Germany; 4 Department of Neuropathology, Berlin Institute of Health (BIH), Charité—Universitätsmedizin, Freie Universität Berlin, Humboldt-Universität Zu Berlin, Berlin, Germany; 5 Department of Neuropathology, Universitätsmedizin—Mainz, Mainz, Germany

The involvement of the complement system in the pathogenesis of myasthenia gravis (MG) depends on the IgG subtype. The serum anti-acetylcholine receptor antibody (AChR-ab, present in about 80% of all MG patients) essentially belongs to the IgG1 subtype and can, therefore, activate the classical complement pathway. In contrast, serum antibodies against the muscle-specific tyrosine kinase (MuSK-ab, present in about 3% of all MG patients) are mostly from the IgG4 subtype that do not activate the complement system [6, 12]. Other previously identified antibodies are directed against the lipoprotein-related protein 4 (LRP4-ab, present in 2% of all MG patients) [2]. Approximately, 15% of MG patients are termed “seronegative” (SNMG), meaning that no known serum antibodies can be detected. Clinicoserological diagnosis alone carries the risk of under-diagnosis, which may exclude SNMG patients from modern therapies: targeted complement inhibition (eculizumab) has recently been introduced in the treatment of AChR-ab-positive generalized MG patients who do not respond to standard treatment [5]. The aim of this study was to identify a reliable biomarker to justify complement-targeting therapies in SNMG.

To investigate the role of the complement system in SNMG, we performed a cross-sectional study in 11 patients with treatment-refractory SNMG who prospectively underwent external intercostal muscle biopsy. Furthermore, we retrospectively analyzed previously performed biopsies of deltoid muscles from two patients with SNMG. Diagnosis of SNMG was established as follows: (i) typical clinical presentation with fatigable muscle weakness that improves with rest and (ii) absence of detectable autoantibodies against AChR, MuSK (measured by enzyme-linked immunosorbent assay, ELISA) and LRP4 (measured by indirect immunofluorescence test, IIFT) in patients’ sera and (iii) abnormal results in repetitive nerve stimulation and/or single-fiber electromyography and/or (iv) clinical response to intravenous or oral acetylcholinesterase inhibitors. Generalized, treatment-refractory disease course was defined as follows: (i) Myasthenia Gravis Foundation of America (MGFA) classification ≥ II despite (ii) standard therapy consisting of acetylcholinesterase inhibitors, steroids, and long-term immunosuppressants and/or (iii) repeated need for intravenous immunoglobulins and/or plasmapheresis/immunoadsorption. Muscle specimens were analyzed by conventional and immunostaining, immunofluorescence and electron microscopy. The results were compared to ‘disease controls’ (i.e. patients with AChR-ab-positive MG) and ‘non-disease controls’ (i.e. patients with non-specific muscle complaints who had no morphological or serological abnormalities). In all patients, stains were done under standardized conditions using the same batches of antibodies. Endplates could be identified in all patients by staining with non-specific esterase (NSE), acetylcholine esterase (AChE) and CD56 (a neural cell adhesion molecule on the pre- and postsynaptic membrane). All patients gave written informed consent. All procedures were approved by the official institutional ethics review committee (EA2/163/17) at the Charité—University Hospital Berlin.

Overall, we included 13 patients with SNMG. Mean age was 44.0 years (SD 19.8), 9 (69.2%) were female. Median disease duration was 6.3 years (SD 5.3). Disease severity ranged from MGFA class IIa–V, eight patients had a history of myasthenic crisis (see Table 1 for a detailed overview of the patient characteristics). In all biopsies of SNMG patients (mean number of endplates per high-power field-analysis [HPF] = 12.7, SD = 9.0), C5b-9 (membrane attack complex, MAC), the terminal complex of the complement pathway, was stained positive on motor endplates. Endplates were also C1q-positive, giving proof of the involvement of the classical pathway of complement activation. Co-localization of C5b-9 with IgG1 revealed direct involvement of G1 immunoglobulins and was consistently double-positive in all analyzed skeletal muscle specimens (Fig. 1). All ‘non-diseased’ controls (*n* = 3, mean number of endplates per HPF = 12.3, SD = 3.5) were negative for IgG1 and C5b-9. Consecutive slices stained for NSE, AchE and CD56 as well as C5b-9 show consistent and specific staining on endplates in AchR-ab-positive and SNMG patients (supplemental figure). Ultrastructural analysis was limited to few endplates in each case and showed no major alterations in the terminal axons or postsynaptic clefts both in seronegative and in AChR-ab-positive biopsies (*n* = 3).

**Table 1:** Patient characteristics

Patient	Age	Sex	Disease duration (years)	Electrophysiological findings (RNS and/or SFEMG)	Response to AchE inhibitors	Thymectomy	Myasthenic crisis	Current MGFA class	Highest MGFA class
I	22	m	6	+	+	+ (no abnormalities)	+	IIIa	V
II	56	m	9	+	+	−	+	IIIb	V
III	72	m	21	+	+	+ (thymic hyperplasia)	+	IIIb	V
IV	49	w	5	+	+	−	−	IIIb	IIIb
V	49	w	6	+	+	+ (no abnormalities)	−	IIIb	IIIb
VI	72	w	2	−	+	−	+	IIIb	V
VII	57	m	7	−	+	−	−	IIb	IIb
VIII	30	w	1	−	+	+ (thymic hyperplasia)	−	IIa	IIa
IX	18	w	5	−	+	+ (no abnormalities)	+	IIIa	V
X	32	w	11	−	+	+ (thymic hyperplasia)	+	IIb	V
XI	29	w	2	−	+	+ (thymic hyperplasia)	−	IIb	
XII	20	w	1	+	+	+ (no abnormalities)	+	IIIb	V
XIII	66	w	6	+	+	+ (no abnormalities)	+	IIIb	V

*RNS* repetitive nerve stimulation, *SFEMG* single-fiber electromyography, *AchE* acetylcholinesterase, *MGFA* Myasthenia gravis Foundation of America classification

**Fig. 1:**
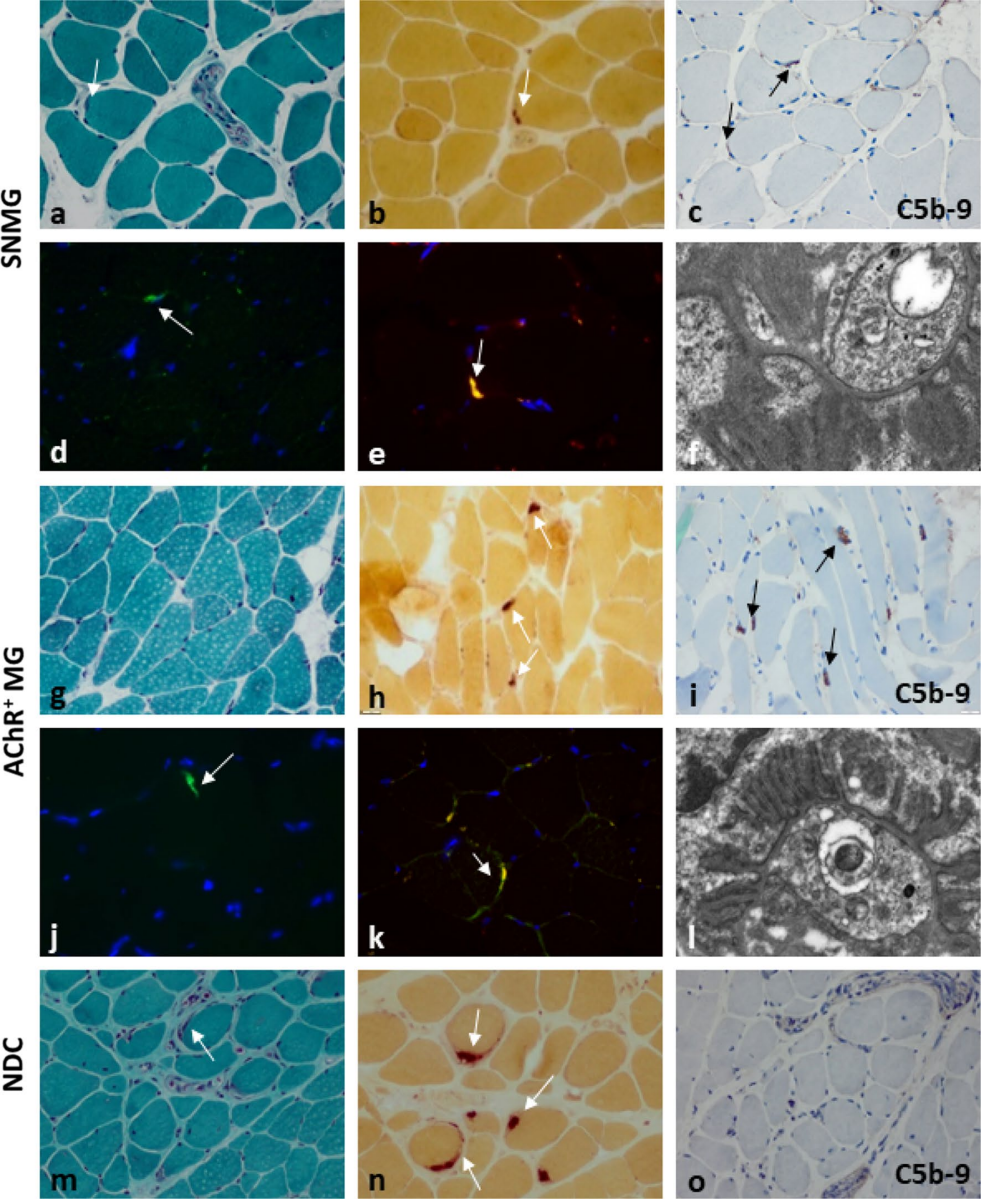
Representative findings in a SNMG patient’s muscle showing **a** an endplate area stained by Gömöri trichrome with fibres standing apart from each other and sometimes small nerve fascicles penetrating, **b** endplates stained for non-specific esterase (NSE, enzyme histochemical staining), endplates positive for **c** C5b-9 (immunehistochemical staining), and **d** C1q (immunofluorescence staining, AF488-direct labelling; green), **e** endplates co-stained for C5b-9 (immunofluorescence staining, AF488; green) and IgG1 (Cy3; red), nuclei are stained with DAPI (blue) **f** no major plump or shortened postsynaptic clefts by electron microscopy (original magnification of × 20.000; all other stains with original magnification of  × 400). Representative findings in an AChR-ab-positive MG patient’s muscle showing **g** an endplate area stained by Gömöri trichrome, **h** endplates stained for NSE endplates, endplates positive for **i** C5b-9 and **j** C1q (AF488; green), **k** endplates, co-stained for C5b-9 (green) and IgG1 (red), nuclei (blue) **l** no major plump or shortened postsynaptic clefts by electron microscopy (original magnification of × 20.000; all other stains with original magnification of × 400). Representative findings of a non-disease control showing **m** an endplate area stained by Gömöri trichrome, **n** endplates stained for NSE **o** endplates without positivity for C5b-9, the endplate area can be clearly identified by the presence of terminal nerve endings. All stains with original magnification of × 400. *SNMG* seronegative myasthenia gravis, *AchR + MG* acetylcholine receptor antibody-positive myasthenia gravis, *NDC* non-diseased controls

To the best of our knowledge, we provide first ‘in situ’ evidence that in skeletal muscle of SNMG patients (i) complement deposits can consistently be identified at motor-endplates, and (ii) that the complement system is activated via the classical IgG1-mediated pathway. Most histopathological studies on myasthenia gravis stem from 1970s to 1980s. The majority of these studies was done in mice with experimental autoimmune MG (EAMG) and showed complement deposits at the neuromuscular junction (NMJ). These findings were partly replicated in MG patients. However, with the anti-AChR-ab being identified in 1976 [7], the histopathological studies either provide no information on the patients’ antibody status at all [1] or only on the presence or absence of AChR-ab [11]. MuSK-ab have been identified in 2001 [4] and LRP-4-ab as late as in 2011 [3]. Hence, the present histopathological analyses are the first being performed in so-called “triple seronegative” MG patients. Antibody testing was performed by ELISA or IIFT. Cell-based assays (CBA) are reported to have a higher sensitivity. However, our approach reflects current clinical practice since the handling and interpretation of CBA need tissue culture facilities and expertise. Hence, their use is currently confined to specialized research centers [8]. In this study, we opted for external intercostal muscle biopsy, since it has been shown to be of unique usefulness in the study of human neuromuscular disease [9, 10]. The external intercostal muscle is a short, flat muscle with abundant motor endplates allowing for detailed examinations of the NMJ. Alternatively, biopsies of limb muscles can be performed but yield the risk of not providing (sufficient) motor endplates within the biopsied area.

In conclusion, the histopathological evidence of an involvement of the complement system in SNMG could be of diagnostic and therapeutic relevance. In the light of a growing trend towards laboratory and instrument-based diagnostics, patients as well as clinicians face an increasing diagnostic uncertainty in cases where “only” the clinical presentation is suggestive of the diagnosis of MG. Thus, biopsy and histopathological analysis of the external intercostal muscles seem to be a feasible diagnostic step in establishing the diagnosis of SNMG, especially in refractory disease courses. Individualized therapies may include targeted complement inhibition in refractory SNMG with histopathologically confirmed complement deposition at the NMJ.

## Electronic supplementary material

Below is the link to the electronic supplementary material.

Supplementary file1 Supplemental figure: Representative consecutive slices stained in a SNMG patient, showing **a** NSE-positive endplates, **b** AchE-positive endplates, **c** CD56-positive endplates, **d** C5b-9-positive endplates. Representative consecutive slices stained in an AChR-ab-positive MG-patient, showing **e** NSE-positive endplates, **f** AchE-positive endplates, **g** CD56-positive endplates, **h** C5b-9-positive endplates. Representative consecutive slices stained in a non-disease control patient, showing **i** NSE-positive endplates, **j** AchE-positive endplates, **k** CD56-positive endplates and **l** C5b-9-stain—no endplates stained. All stains with original magnification of × 400. *SNMG* seronegative myasthenia gravis, *AchR + MG* acetylcholine receptor antibody-positive myasthenia gravis, *NDC* non-diseased controls, *NSE* non-specific esterase, *AchE* acetylcholine esterase (TIF 1379 kb)
