# Supplementary figures and images for: Complement deposition at the neuromuscular junction in seronegative myasthenia gravis
Source: Acta Neuropathol. 2020 Mar 10;139(6):1119–22. doi: 10.1007/s00401-020-02147-5 (PMC7244604; doi:10.1007/s00401-020-02147-5)

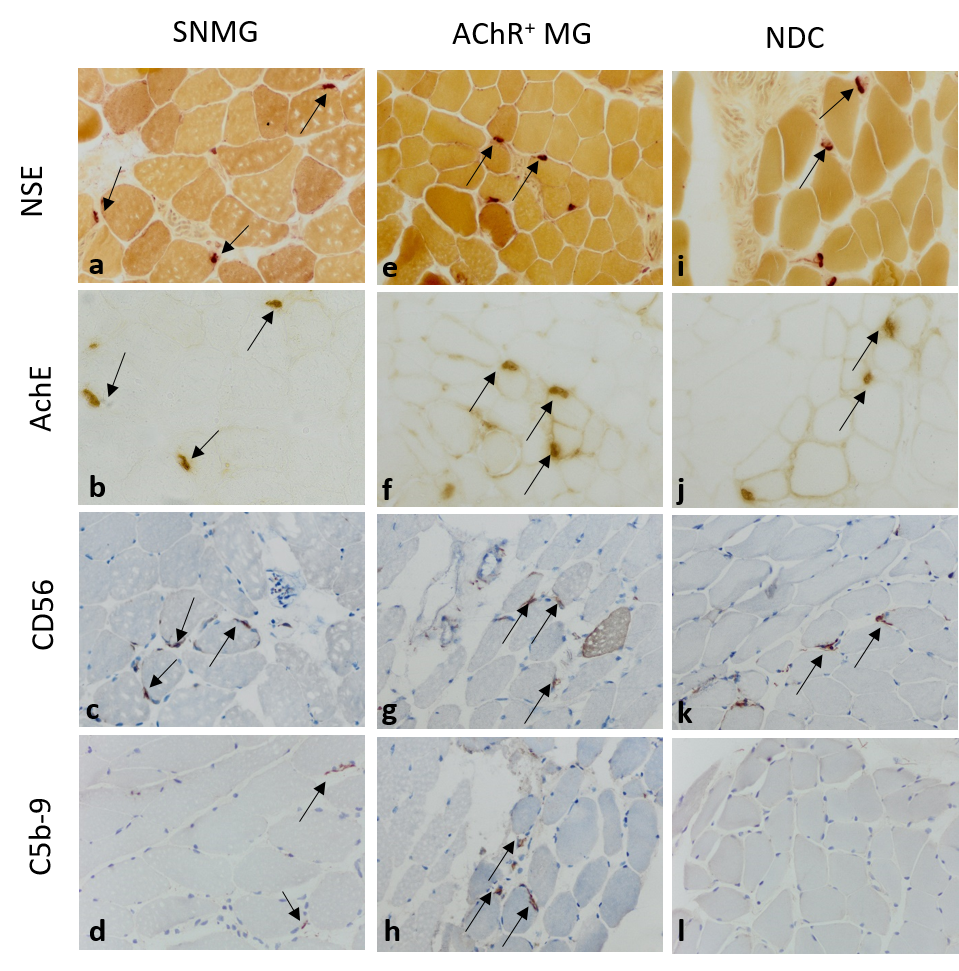

Supplement: Supplementary file 1 — Supplementary file1 Supplemental figure: Representative consecutive slices stained in a SNMG patient, showing a NSE-positive endplates, b AchE-positive endplates, c CD56-positive endplates, d C5b-9-positive endplates. Representative consecutive slices stained in an AChR-ab-positive MG-patient, showing e NSE-positive endplates, f AchE-positive endplates, g CD56-positive endplates, h C5b-9-positive endplates. Representative consecutive slices stained in a non-disease control patient, showing i NSE-positive endplates, j AchE-positive endplates, k CD56-positive endplates and l C5b-9-stain—no endplates stained. All stains with original magnification of × 400. SNMG seronegative myasthenia gravis, AchR + MG acetylcholine receptor antibody-positive myasthenia gravis, NDC non-diseased controls, NSE non-specific esterase, AchE acetylcholine esterase (TIF 1379 kb) [file 401_2020_2147_MOESM1_ESM.tif]
